# Isoquercitrin Delays Denervated Soleus Muscle Atrophy by Inhibiting Oxidative Stress and Inflammation

**DOI:** 10.3389/fphys.2020.00988

**Published:** 2020-08-12

**Authors:** Yuntian Shen, Qiuyu Zhang, Ziwei Huang, Jianwei Zhu, Jiayi Qiu, Wenjing Ma, Xiaoming Yang, Fei Ding, Hualin Sun

**Affiliations:** ^1^ Key Laboratory of Neuroregeneration of Jiangsu and Ministry of Education, Jiangsu Clinical Medicine Center of Tissue Engineering and Nerve Injury Repair, Co-Innovation Center of Neuroregeneration, Nantong University, Nantong, China; ^2^ Department of Orthopedics, Affiliated Hospital of Nantong University, Nantong, China; ^3^ School of Nursing, Nantong University, Nantong, China

**Keywords:** denervated muscle atrophy, isoquercitrin, oxidative stress, inflammation, proteolysis, mitophagy

## Abstract

Although denervated muscle atrophy is common, the underlying molecular mechanism remains unelucidated. We have previously found that oxidative stress and inflammatory response may be early events that trigger denervated muscle atrophy. Isoquercitrin is a biologically active flavonoid with antioxidative and anti-inflammatory properties. The present study investigated the effect of isoquercitrin on denervated soleus muscle atrophy and its possible molecular mechanisms. We found that isoquercitrin was effective in alleviating soleus muscle mass loss following denervation in a dose-dependent manner. Isoquercitrin demonstrated the optimal protective effect at 20 mg/kg/d, which was the dose used in subsequent experiments. To further explore the protective effect of isoquercitrin on denervated soleus muscle atrophy, we analyzed muscle proteolysis *via* the ubiquitin-proteasome pathway, mitophagy, and muscle fiber type conversion. Isoquercitrin significantly inhibited the denervation-induced overexpression of two muscle-specific ubiquitin ligases—muscle RING finger 1 (MuRF1) and muscle atrophy F-box (MAFbx), and reduced the degradation of myosin heavy chains (MyHCs) in the target muscle. Following isoquercitrin treatment, mitochondrial vacuolation and autophagy were inhibited, as evidenced by reduced level of autophagy-related proteins (ATG7, BNIP3, LC3B, and PINK1); slow-to-fast fiber type conversion in the target muscle was delayed *via* triggering expression of peroxisome proliferator-activated receptor γ coactivator 1α (PGC-1α); and the production of reactive oxygen species (ROS) in the target muscle was reduced, which might be associated with the upregulation of antioxidant factors (SOD1, SOD2, NRF2, NQO1, and HO1) and the downregulation of ROS production-related factors (Nox2, Nox4, and DUOX1). Furthermore, isoquercitrin treatment reduced the levels of inflammatory factors—interleukin (IL)-1β, IL-6, and tumor necrosis factor-α (TNF-α)—in the target muscle and inactivated the JAK/STAT3 signaling pathway. Overall, isoquercitrin may alleviate soleus muscle atrophy and mitophagy and reverse the slow-to-fast fiber type conversion following denervation *via* inhibition of oxidative stress and inflammatory response. Our study findings enrich the knowledge regarding the molecular regulatory mechanisms of denervated muscle atrophy and provide a scientific basis for isoquercitrin as a protective drug for the prevention and treatment of denervated muscle atrophy.

## Introduction

Skeletal muscle, an integral part of the human body, is involved in various bodily functions including exercise, assisted breathing, heat production, internal organ protection, and glucose and fat metabolism. Skeletal muscle comprises approximately 40% of the total body weight. Maintenance of skeletal muscle homeostasis is essential for maintaining the body’s various functions (Ferreira et al., 2019; Yamakawa et al., 2020). An imbalance in skeletal muscle homeostasis can cause skeletal muscle hyperplasia or atrophy. Skeletal muscle atrophy is characterized by reduced muscle mass and muscle fibers, weakened muscle strength, and functional decline. Patients with severe muscle atrophy can lose the ability to move and are bedridden. Long-term bedridden conditions aggravate muscle atrophy and cause various life-threatening complications (Ceco et al., 2017; Sakellariou and McDonagh, 2018). Skeletal muscle atrophy commonly occurs concomitant to various conditions, including peripheral nerve injury, weightlessness, limb immobilization, aging, and numerous diseases (e.g., cancer cachexia, diabetes, heart failure, and kidney failure; Hyatt et al., 2019; Salucci and Falcieri, 2020).

Peripheral nerve injury is a common disease that inevitably causes a certain degree of neuronal degeneration, muscle atrophy, and fibrosis. During the repair of long-distance nerve defects, target muscle atrophy is often irreversible because the regenerated nerve grows gradually and is unable to reach the target muscle in time. Consequently, muscle atrophy critically affects the functional reconstruction of the target muscle, placing a substantial burden on the patient’s family and on society (Tuffaha et al., 2016). Therefore, promoting nerve regeneration ability and delaying skeletal muscle atrophy progression are crucial for improving the functional reconstruction of the injured nerve (Tos et al., 2013; Chiono and Tonda-Turo, 2015). Although a substantial amount of research on denervated muscle atrophy has been conducted, a sound method for the prevention and treatment of denervated muscle atrophy is lacking, which may be owing to a poor understanding of the molecular mechanisms involved.

Muscle atrophy is characterized by a decreased contractility of muscle fibers, changes in muscle fiber types and myosin subtypes, and a net loss of cytoplasm, organelles, and total proteins (Dumitru et al., 2018). In recent years, although research on denervated muscle atrophy has made substantial progress, it has mainly focused on single events, genes, or proteins (Li et al., 2017; Reza et al., 2017; Gueugneau et al., 2018; Yin et al., 2018; Castets et al., 2019; Janice Sánchez et al., 2019). Because systematic research on denervated muscle atrophy has not yet been conducted, no breakthrough has been achieved. Numerous molecules involved in denervated muscle atrophy are also involved in several events or pathways that are interconnected, including proteolytic pathways (such as the ubiquitin-proteasome pathway and autophagy-lysosomal pathway), protein synthesis pathways, and muscle fiber regeneration pathways (Winbanks et al., 2016; Arouche-Delaperche et al., 2017; Li et al., 2017; Quattrocelli et al., 2017; Brzeszczyńska et al., 2018; Yin et al., 2018; Huang et al., 2019; Qiu et al., 2019; Wu et al., 2019). Moreover, interconnections between these pathways complicate the molecular mechanism of denervated muscle atrophy. Therefore, it is crucial to identify targets that can effectively delay the process of muscle atrophy, emphasizing on the search for an upstream factor or event that initially triggers muscle atrophy, to provide new strategies for the prevention and treatment of denervated muscle atrophy.

Considering this scenario, our research group has performed a series of studies on denervated muscle atrophy using genomics and proteomics. We have found that proteolysis *via* the ubiquitin-proteasome and autophagy-lysosomal proteolytic athways and the protein synthesis pathway plays an important role in the process of denervated muscle atrophy (Sun et al., 2006, 2009, 2012, 2014a,b; He et al., 2016; Qiu et al., 2018). Recently, we used transcriptome sequencing and bioinformatics methods to systematically analyze the differentially expressed genes involved in denervated muscle atrophy. To the best of our knowledge, it was proposed for the first time that denervated muscle atrophy can be divided into four different transcription stages (Shen et al., 2019). In the oxidative stress stage (0–12 h), oxidative stress occurs early after skeletal muscle denervation, which may be attributed to the loss of contractile function of the target muscle, resulting in decreased blood perfusion in the muscle. Therefore, under a relatively hypoxic state, reactive oxygen species (ROS) will be produced. Persistent hypoxia results in excessive ROS production, causing an imbalance between the oxidation and antioxidation systems. In the inflammation stage (24 h), excessive ROS production causes tissue damage, thereby inducing the generation of numerous inflammatory factors. These inflammatory factors cause further inflammation by activating inflammatory response pathways. In the atrophic (3–7 days) and atrophic fibrosis (14–28 days) stages, excessively activated inflammation further initiates the downstream muscle atrophic process, thereby promoting target muscle atrophy and fibrosis (Shen et al., 2019). Overall, oxidative stress, inflammation, atrophy, and atrophic fibrosis sequentially occur following skeletal muscle denervation. Previous studies have reported that high ROS levels can cause proteolysis, muscle cell apoptosis, and eventual skeletal muscle atrophy (Theilen et al., 2017; Ábrigo et al., 2018; Guigni et al., 2018). Muller et al. (2007) found a significant increase in ROS levels in the aging and skeletal muscles of patients with amyotrophic lateral sclerosis, and the ROS level was closely related to the degree of skeletal muscle atrophy. Other studies have indicated that inflammation is involved in skeletal muscle atrophy caused by tumor cachexia and sepsis (Zhu et al., 2017; Cerquone Perpetuini et al., 2018). These results suggest that oxidative stress and inflammatory signals that are sequentially activated within 24 h following skeletal muscle denervation are essential for triggering denervated skeletal muscle atrophy.

Isoquercitrin (quercetin-3-O-β-D-glucopyranoside) is a flavonoid compound widely distributed in plants. It possesses various biological properties, such as anti-inflammatory, antioxidative, anti-allergic, and anti-viral activities (Li et al., 2019). Existing studies have found that isoquercitrin exerts a neuroprotective effect on ischemic stroke. By activating the nuclear factor erythroid 2-related factor 2 (Nrf2) pathway, isoquercitrin promotes the expression of antioxidant enzymes, thereby inhibiting the NOX4/ROS/nuclear factor κB (NF-κB) pathway and reducing oxidative stress and neuronal apoptosis (Dai et al., 2018). Reportedly, isoquercitrin improves the production of inflammatory factors, including tumor necrosis factor-α (TNF-α), interleukin (IL)-1β, and IL-6, by blocking the NF-κB and mitogen-activated protein kinase (MAPK) pathways for protecting the liver from acetaminophen-induced damage (Xie et al., 2016), reduces high-fat diet- and beta-amyloid-induced oxidative stress for improving cognitive function in mice (Kim et al., 2019), and upregulates Nrf2 expression, inhibits the NF-kB pathway, and regulates the AMP-activated protein kinase pathway to alleviate streptozotocin-induced diabetic symptoms in rats (Jayachandran et al., 2019). However, studies on whether isoquercitrin can alleviate muscle atrophy and the underlying molecular mechanisms are lacking.

Therefore, in the present study, we established a denervated muscle atrophy model using sciatic nerve disruption in mice, followed by intraperitoneal injection. The atrophy of soleus muscle, containing 98% slow type muscle fibers, was greater affected by sciatic nerve transection as compared to tibialis anterior and extensor digitorum longus muscles (Beehler et al., 2006; Higashino et al., 2013). The wet weight ratio, muscle fiber cross-sectional area (CSA), mitophagy, and muscle fiber type conversion of the soleus muscle were investigated to evaluate the protective effect of isoquercitrin on denervated muscle atrophy. Expression of genes and proteins related to inflammation and oxidative stress was determined to analyze the possible mechanism *via* which isoquercitrin delays denervated muscle atrophy. This study attempted to further enrich the knowledge regarding the molecular mechanism of denervated muscle atrophy and to provide a scientific basis for isoquercitrin as a protective drug for the prevention and treatment of muscle atrophy.

## Materials and Methods

### Animal Experiment

Healthy (6–8 weeks old) male Institute of Cancer Research (ICR) mice (weight, 20 ± 2 g) were provided by the Experimental Animal Center of Nantong University, China. The experiments involving animals were carried out in accordance with the animal care guidelines of Nantong University and ethically approved by Jiangsu Administration Committee of Experimental Animals. The mice were anesthetized using an intraperitoneal injection of a compound anesthetic, and the sciatic nerve was exposed and further isolated. A 1-cm sciatic nerve transection was made in the femoral segment of the left hind limb, which was then disinfected and sutured. The mice were randomized into the following groups: sham operation group (Ctrl), denervation group (DEN), and denervation + isoquercitrin (10, 20, and 40 mg/kg/d) group (ISO-L, ISO-M, and ISO-H). Isoquercitrin or saline was administrated by intraperitoneal injection after sciatic nerve transection and lasted for 14 consecutive days in each group, respectively. Thereafter, the mice were killed, and the bilateral soleus muscles of the hind limbs of each mouse were collected, weighed, and stored using different storage methods as per the requirements of subsequent experiments. The wet weight ratio of muscle was calculated by the injury side compared with the contralateral side.

### Immunofluorescence Staining

Following sample fixation with 4% paraformaldehyde, the entire soleus muscle was removed and dehydrated in a series of 10, 20, and 30% sucrose. Thereafter, one-third of the muscles were embedded in optimal cutting temperature (OCT) compound, frozen, and sliced into 8-μm-thick sections. These sections were mounted and incubated overnight with primary antibodies for laminin or fast myosin skeletal heavy chain (Abcam, Cambridge, UK). On the following day, the sections were rinsed with phosphate-buffered saline (PBS), incubated with the corresponding secondary antibodies at room temperature for 1–2 h, rinsed with PBS, mounted, dried, and photographed using a Zeiss fluorescent microscope.

### Dihydroethidium Probe for Determination of ROS Level

The mice were irrigated with saline at room temperature, followed by perfusion with dihydroethidium (DHE) solution (10 μM, Beyotime, Haimen, China) for 1 h. Thereafter, they were perfused with 4% paraformaldehyde, following which the soleus muscles were removed and dehydrated in a series of 10, 20, and 30% sucrose. One-third of the soleus muscles obtained was embedded in OCT compound, and 8-μm-thick frozen sections were prepared and directly observed using the Zeiss fluorescent microscope.

### Quantitative Reverse Transcription-Polymerase Chain Reaction

Using quantitative reverse transcription–polymerase chain reaction (qRT-PCR), levels in messenger RNAs (mRNAs) were analyzed, as described (Huang et al., 2019). In short, total RNA was extracted from soleus muscle samples and used to generate cDNA samples. PCR was performed according to our previous study. The primers were as follows: mouse *Nrf2* Forward: TAGATGACCATGAGTCGCTTGC, Reverse: GCCAAACTTGCTCCATGTCC; mouse *NQO1* Forward: AGGATGGGAGGTACTCGAATC, Reverse: TGCTAGAGATGACTCGGAAGG; mouse *HO-1* Forward: AGGTACACATCCAAGCCGAGA, Reverse: CATCACCAGCTTAAAGCCTTCT; mouse *IL-6* Forward: CTGCAAGAGACTTCCATCCAG, Reverse: AGTGGTATAGACAGGTCTGTTGG, mouse *IL-1β* Forward: GAAATGCCACCTTTTGACAGTG, Reverse: TGGATGCTCTCATCAGGACAG; mouse *TNF-α* Forward: CAGGCGGTGCCTATGTCTC, Reverse: CGATCACCCCGAAGTTCAGTAG; mouse *SOD1* Forward: AACCAGTTGTGTTGTCAGGAC, Reverse: CCACCATGTTTCTTAGAGTGAGG; mouse *SOD2* Forward: CAGACCTGCCTTACGACTATGG, Reverse: CTCGGTGGCGTTGAGATTGTT; mouse *Duox1* Forward: TATCTCCCCAGAGTTCGTTGT, Reverse: GGGTGCTCTCGACTCCAGT; mouse *GAPDH* Forward: AACTTTGGCATTGTGGAAGG, Reverse: ACACATTGGGGGTAGGAACA; The relative mRNA expression was calculated by the 2^−ΔΔCt^ method (Livak and Schmittgen, 2001), and glyceraldehyde-3-phosphate dehydrogenase (GAPDH) level was used as the internal control.

### Western Blot

The total protein in the muscle was extracted from the protein lysate. Protein concentration was measured using a BCA kit (Beyotime, Haimen, China). Thereafter, 30 μg of the total protein was separated by sodium dodecyl sulfate-polyacrylamide gel electrophoresis and transferred onto a polyvinylidene difluoride (PVDF) membrane *via* the wet transfer method. The PVDF membrane was blocked for 1 h, followed by incubation with the primary antibody antibodies: mouse anti-MyHC (R&D Systems, Minneapolis, MN), rabbit anti-NOX4 (Invitrogen, Rockford, IL, USA), rabbit anti-MAFbx, rabbit anti-PINK1, rabbit anti-ATG7, mouse anti-BNIP3, rabbit anti-LC3B, rabbit anti-NOX2, rabbit anti-Nrf2, rabbit anti-NQO1, rabbit anti-PGC-1α (Abcam, Cambridge, UK), mouse anti-p-Jak1 (Tyr1034/1035)/Jak2 (Tyr1007/1008), rabbit anti-p-Stat3 (Tyr705) and rabbit anti-Stat3 (Cell Signaling technology, MA, USA), rabbit anti-MuRF-1, mouse anti-Troponin I-FS, mouse anti-Troponin I-SS, and mouse anti-tubulin (Santa Cruz, Santa Cruz, CA) at 4°C overnight. On the following day, the PVDF membrane was rinsed thrice with tris-buffered saline with Tween (TBST) and incubated with the corresponding secondary antibody at room temperature for 1 h. Thereafter, PVDF membrane was rinsed thrice with TBST, treated with the appropriate amount of luminescent liquid, and finally scanned using a membrane scanner.

### Transmission Electron Microscopy Analysis

To observe the changes in the mitochondria, the soleus muscle was analyzed through transmission electron microscopy (TEM) analysis, as previously reported (Huang et al., 2019). Briefly, 1-mm^3^-sized muscle was fixed in 2.5% glutaraldehyde, followed by post fixation in 1% osmium tetroxide. Muscle sections (20 fields per mouse and three mouse per group) were analyzed by TEM (HT7700, Hitachi, Tokyo, Japan). The number of vacuoles or autophagosomes per 100 mitochondria was calculated.

### Enzyme-Linked Immunosorbent Assay

To observe the changes of proinflammatory cytokines, the content of IL-1β, IL-6, and TNF-α was measured according to the manufacturer’s instructions. Briefly, enzyme-linked immunosorbent assay (ELISA) plates (Beyotime, Haimen, China) were incubated with 100 μl muscle lysates at 37°C for 2 h, followed by incubation with anti-IL-1β, anti-IL-6, or anti-TNF-α antibodies for 1 h. Subsequently, ELISA plates were washed and incubated with horseradish peroxidase (HRP)-streptavidin for 20 min. Absorbance (450 nm) was measured using a microplate spectrophotometer.

### Statistical Analysis

Data in this study were analyzed using one-way ANOVA, followed by the Tukey’s multiple comparisons test. All statistical analyses were conducted with GraphPad Prism software (version 7.0; San Diego, CA, USA). The level of significance was set at *p* < 0.05.

## Results

### Isoquercitrin Relieves Skeletal Muscle Atrophy Caused by Denervation

**Figure 1:**
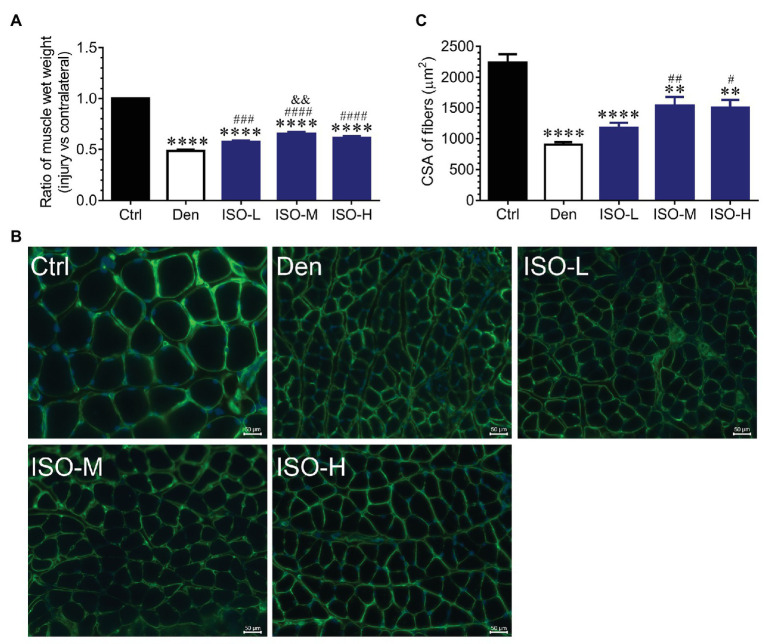
Isoquercitrin reduces soleus muscle mass loss caused by denervation (*n* = 6). **(A)** The ratio of muscles wet weight in each group. **(B)** Representative images of laminin-stained muscles cross-sections in each group. Green indicates laminin staining. Scale bar: 50 μm. **(C)** The histogram shown the cross-sectional area (CSA) of muscles in each group. Ctrl, control group; Den, denervation group; ISO-L, denervated target muscle plus low-dose isoquercitrin (10 mg/kg/d); ISO-M, denervated target muscle plus middle-dose isoquercitrin (20 mg/kg/d); ISO-H, denervated target muscle plus high-dose isoquercitrin (40 mg/kg/d). ^**^p < 0.01 and ^****^p < 0.0001 vs. Ctrl; ^#^*p* < 0.05, ^##^*p* < 0.01, ^###^*p* < 0.001, and ^####^*p* < 0.0001 vs. Den; ^&&^*p* < 0.01 vs. ISO-L.

We used ICR adult mice to prepare sciatic nerve transection models. The mice were randomly divided into sham operation, DEN, low-dose isoquercitrin (10 mg/kg/d), middle-dose isoquercitrin (20 mg/kg/d), and high-dose isoquercitrin (40 mg/kg/d) groups. The soleus muscle of each mouse was obtained after 2 weeks of treatment, and the wet weight ratio of the soleus muscle was analyzed. The target muscle wet weight ratio in the DEN was significantly lower than that in the control group (*p* < 0.001), indicating that the denervated muscle atrophy model was successfully prepared. The target muscle wet weight ratios in the isoquercitrin-treated groups were significantly higher than that in DEN, indicating that isoquercitrin alleviates skeletal muscle atrophy caused by denervation in a dose-dependent manner. Middle-dose isoquercitrin (20 mg/kg/d) demonstrated the optimal protective effect in the mouse model of denervated muscle atrophy (Figure 1).

Laminin immunofluorescence staining performed to further investigate the effect of isoquercitrin on denervated muscle atrophy revealed that the muscle fiber cross-sectional area in DEN was significantly smaller than that in the control group, indicating that denervation significantly reduced the muscle fiber cross-sectional area. Compared with DEN, the muscle fiber cross-sectional area in the low-dose isoquercitrin group was larger but not of significance, whereas the muscle fiber cross-sectional area in the middle- and high-dose isoquercitrin group was significantly larger (*p* < 0.01). It was suggested that isoquercitrin inhibits the reduction in the muscle fiber cross-sectional area caused by denervation, and the middle-dose isoquercitrin showed the optimal protective effect (Figure 1). These findings were consistent with the results of the target muscle wet weight ratio, indicating that isoquercitrin intervention can effectively alleviate denervated muscle atrophy.

### Isoquercitrin Inhibits Proteolysis *via* the Ubiquitin-Proteasome Pathway

**Figure 2:**
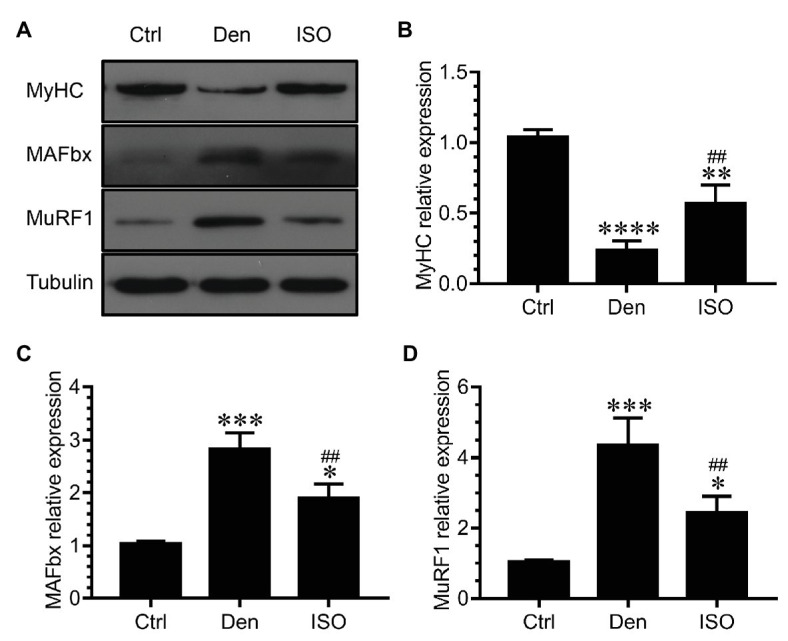
Isoquercitrin inhibits proteolysis *via* the ubiquitin-proteasome pathway (*n* = 6). **(A)** Western blot of MyHC, MAFbx, and MuRF1. **(B–D)** Quantification of the expression of MyHC, MAFbx, and MuRF1. Ctrl, control group; Den, denervation group; ISO, denervated target muscle plus middle-dose isoquercitrin (20 mg/kg/d) group. ^*^p < 0.05, ^**^p < 0.01, ^***^p < 0.001, and ^****^p < 0.0001 vs. Ctrl; ^##^*p* < 0.01vs. Den. MyHC, myosin heavy chain; MuRF1, muscle RING finger 1; MAFbx, muscle atrophy F-box.

Because middle-dose isoquercitrin demonstrated the best effect for preventing and treating denervated muscle atrophy, it was used in subsequent experiments. We measured the expression of the ubiquitin-proteasome proteolytic system during denervated muscle atrophy to further explore the possible mechanism of isoquercitrin in alleviating this condition. The ubiquitin-proteasome system plays an important role in various muscle atrophies (Winbanks et al., 2016; Quattrocelli et al., 2017; Qiu et al., 2019). Our study found that the expression of the muscle-specific ubiquitin ligases muscle atrophy F-box (MAFbx) and muscle RING finger 1 (MuRF1) in DEN was significantly higher than that in the control group. Therefore, the ubiquitin-proteasome proteolytic system in the target muscle was significantly activated following denervation, thereby enhancing the protein degradation ability. In contrast, isoquercitrin treatment significantly reduced the expression of MAFbx and MuRF1 in the denervated muscle and inhibited the activation of the ubiquitin-proteasome proteolytic system in the target muscle following denervation, thereby reducing the proteolytic capacity. Myosin is the main contractile and regulatory protein in skeletal muscle fibers, and myosin heavy chain (MyHC), an important component of myosin, can be used to determine the extent of muscle protein degradation (Tajsharghi and Oldfors, 2013). In the present study, the expression of MyHC in DEN was significantly lower than that in the control group, whereas its expression in the isoquercitrin-treated groups was significantly higher than that in DEN (Figure 2), which may be attributed to the changes in the expression of MAFbx and MuRF1. These findings indicated that isoquercitrin can delay denervated skeletal muscle atrophy progression by inhibiting proteolysis *via* the ubiquitin-proteasome pathway and by reducing myosin degradation.

### Isoquercitrin Reduces Mitophagy Caused by Skeletal Muscle Denervation

**Figure 3:**
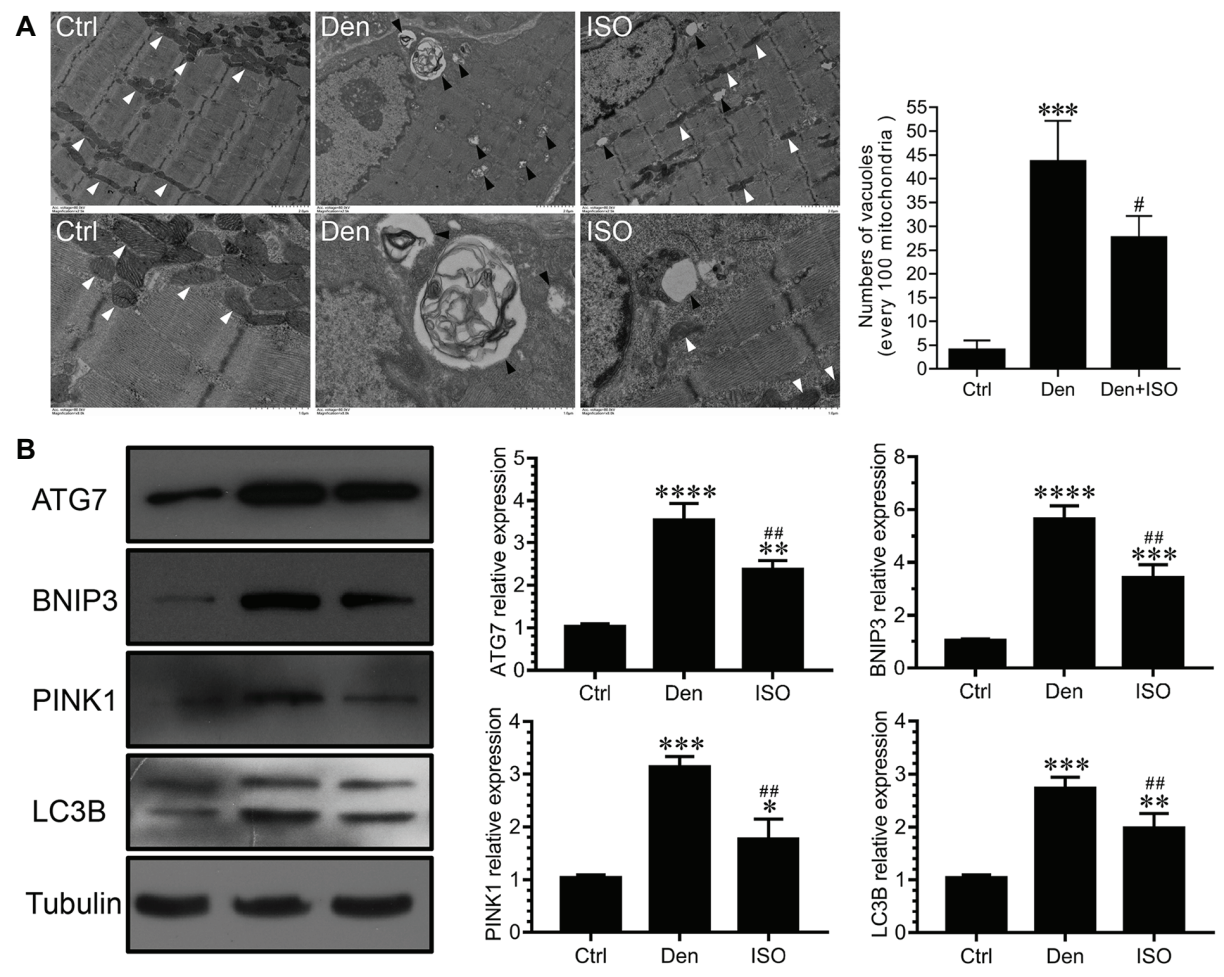
Isoquercitrin reduces mitochondrial autophagy in denervated soleus muscles. **(A)** Ultrastructure of muscle fibers observed using transmission electron microscopy (*n* = 3). The white arrow indicates mitochondria between muscle fibers. The black arrow indicates an autophage or an autophagic vesicle. **(B)** Western blot and quantification of the autophagy-related proteins ATG7, BNIP3, PINK1, and LC3B (*n* = 6). Ctrl, control group; Den, denervation group; ISO, denervated target muscle plus isoquercitrin (20 mg/kg/d) group. ^*^p < 0.05, ^**^p < 0.01, ^***^p < 0.001, and ^****^p < 0.0001 vs. Ctrl; ^#^*p* < 0.05 and ^##^*p* < 0.01 vs. Den.

Studies have shown that autophagy plays an important role in the muscle atrophic process (Milan et al., 2015). In our study, the number of vacuoles in the DEN was significantly higher than that in the control group, which indicated that vacuolar degeneration and autophagy were observed in a large number of mitochondria following target muscle denervation, accompanied by the high expression of the autophagy-related proteins ATG7, BNIP3, PINK1, and LC3B (Figure 3). However, isoquercitrin treatment significantly inhibited mitochondrial vacuolar degeneration and autophagy and downregulated the expression of ATG7, BNIP3, PINK1, and LC3B (Figure 3). These findings suggested that isoquercitrin alleviates mitophagy by downregulating the expression of autophagy-related proteins in denervated target muscles, thereby delaying denervated muscle atrophy.

### Isoquercitrin Delays the Slow-to-Fast Fiber Type Conversion Caused by Denervation

**Figure 4:**
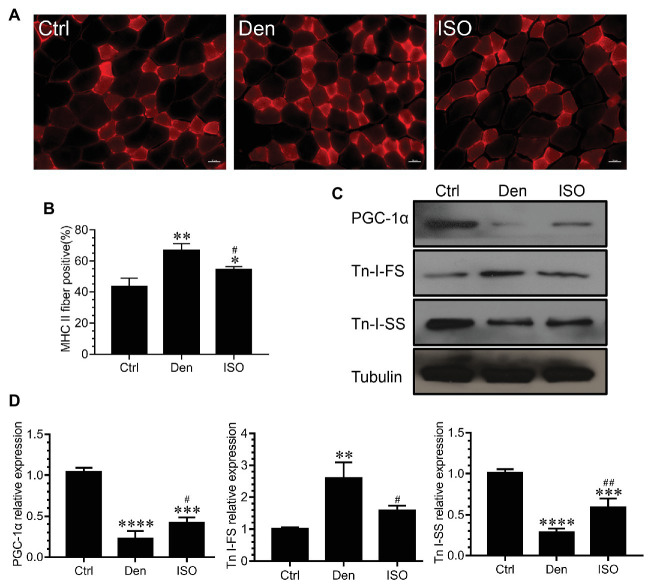
Isoquercitrin delays the slow-to-fast fiber type conversion caused by denervation. **(A)** Immunofluorescence staining of fast myosin skeletal heavy chain in soleus muscle. **(B)** Quantification of the positive proption of fast muscle fibers (*n* = 3). **(C)** Western blot of PGC-1α, Tn I-FS, and Tn I-SS related to the slow-to-fast fiber type conversion. **(D)** Quantification of PGC-1α, Tn I-FS, and Tn I-SS. Ctrl, control group; Den, denervation group; ISO, denervated target muscle plus isoquercitrin (20 mg/kg/d) group. ^*^p < 0.05, ^**^p < 0.01, ^***^p < 0.001, and ^****^p < 0.0001 vs. Ctrl; ^#^*p* < 0.05 and ^##^*p* < 0.01 vs. Den. PGC-1α, peroxisome proliferator-activated receptor γ coactivator 1α; TnI-FS, fast skeletal muscle troponin I; TnI-SS, slow skeletal muscle troponin I.

The conversion between muscle fiber types is an important feature of muscle atrophy. Studies have reported that denervation-induced slow-to-fast fiber type conversion severely impacts the normal function of the target muscle. Immunofluorescence staining of fast muscle fiber protein indicated that the proportion of fast muscle fibers in the soleus muscle following denervation was significantly higher than that in the control group. Moreover, western blots revealed that the expression of fast skeletal muscle troponin I (TnI-FS) significantly increased and that of slow skeletal muscle troponin I (TnI-SS) significantly decreased in the soleus muscle following denervation, suggesting the conversion from slow to fast muscle fibers following denervation. After isoquercitrin intervention, the proportion of fast muscle fibers in the denervated target muscle was significantly decreased, accompanied by TnI-FS downregulation and TnI-SS upregulation. Therefore, isoquercitrin could delay the slow-to-fast fiber type conversion caused by denervation. Peroxisome proliferator-activated receptor γ coactivator 1-alpha (PGC-1α) is a key factor that regulates mitochondrial function, regulates muscle fiber types, and controls the fast-to-slow muscle fiber type conversion (Lin et al., 2002). In the present study, isoquercitrin treatment significantly inhibited a reduction in the expression of PGC-1α in denervated target muscles (Figure 4). These results suggested that isoquercitrin reverses the slow-to-fast fiber type conversion by promoting the expression of PGC-1α, thereby protecting denervated muscle atrophy.

### Isoquercitrin Inhibits the Inflammatory Response Caused by Target Muscle Denervation

**Figure 5:**
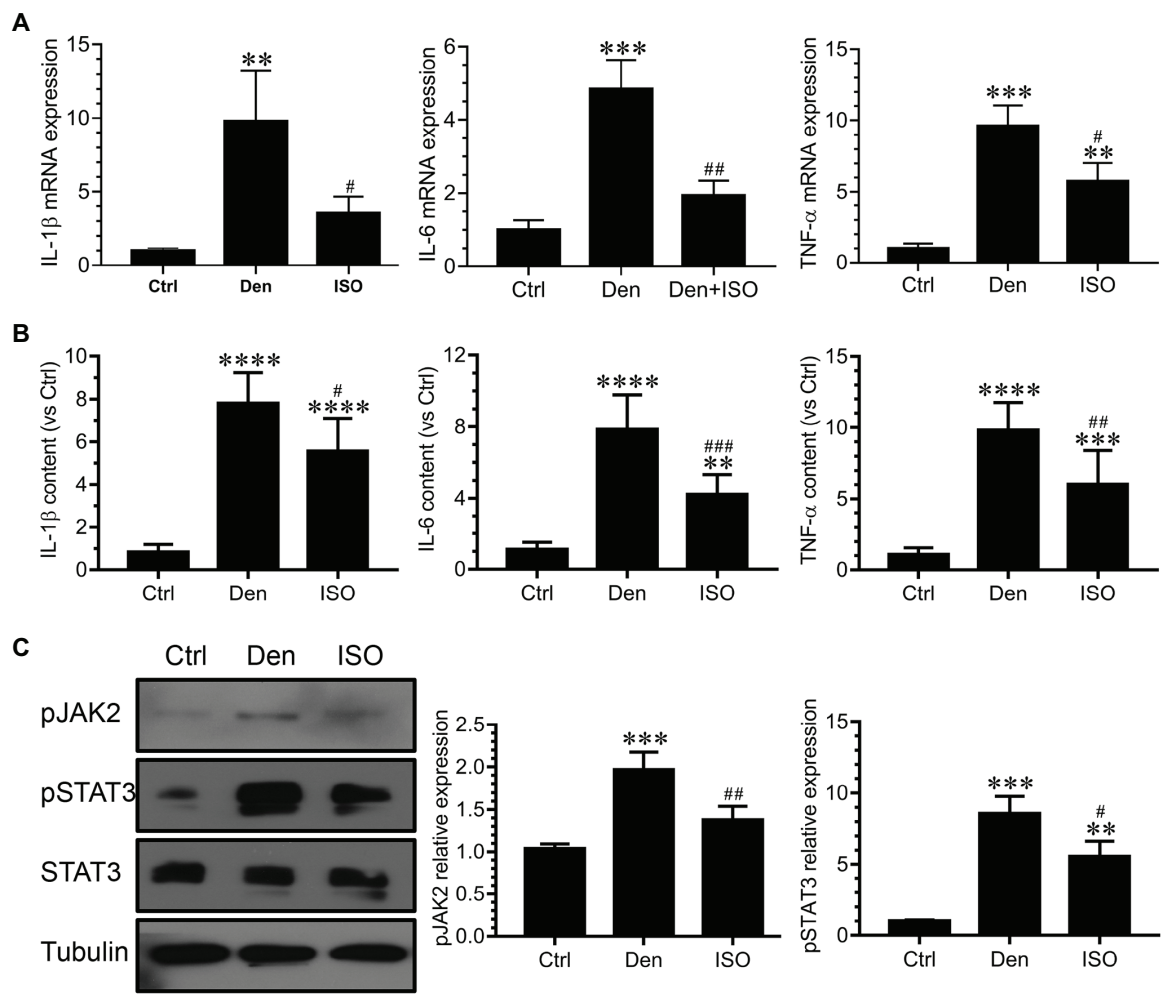
Isoquercitrin inhibits the inflammatory response in denervated soleus muscles (*n* = 6). **(A)** Quantitative polymerase chain reaction detection of changes in the expression of the inflammatory factors interleukin (IL)-1β, IL-6, and tumor necrosis factor (TNF)-α. **(B)** Enzyme-linked immunosorbent assay (ELISA) detection of the expression of IL-1β, IL-6, and TNF-α; **(C)** Western blot analysis of JAK/STAT3 activation. Ctrl, control group; Den, denervation group; ISO, denervated target muscle plus isoquercitrin (20 mg/kg/d) group. ^**^p < 0.01, ^***^p < 0.001, and ^****^p < 0.0001 vs. Ctrl; ^#^*p* < 0.05, ^##^*p* < 0.01, and ^###^*p* < 0.001 vs. Den.

Inflammation is involved in skeletal muscle atrophy caused by tumor cachexia and sepsis, and inflammation inhibition can alleviate muscle atrophy (Zhu et al., 2017; Cerquone Perpetuini et al., 2018). However, it is unclear whether isoquercitrin activity can delay denervated muscle atrophy by suppressing inflammation. Using qRT-PCR and ELISA, we observed that the expression of inflammation-related genes and proteins (IL-1β, IL-6, and TNF-α) in denervated target muscles was significantly increased, suggesting that these inflammatory factors are involved in denervated muscle atrophy. Isoquercitrin treatment significantly inhibited the elevation of the inflammatory factors IL-1β, IL-6, and TNF-α in denervated target muscles (Figures 5A,B). JAK/STAT3 is a classic signaling pathway downstream of IL-6. We observed that although pJAK2, pSTAT3, and STAT3 were significantly overexpressed in denervated target muscles, isoquercitrin could significantly inactivate the JAK2/STAT3 signals (Figure 5C). These results revealed that isoquercitrin may alleviate denervated muscle atrophy by relieving inflammation.

### Isoquercitrin Inhibits Oxidative Stress in Denervated Target Muscles

**Figure 6:**
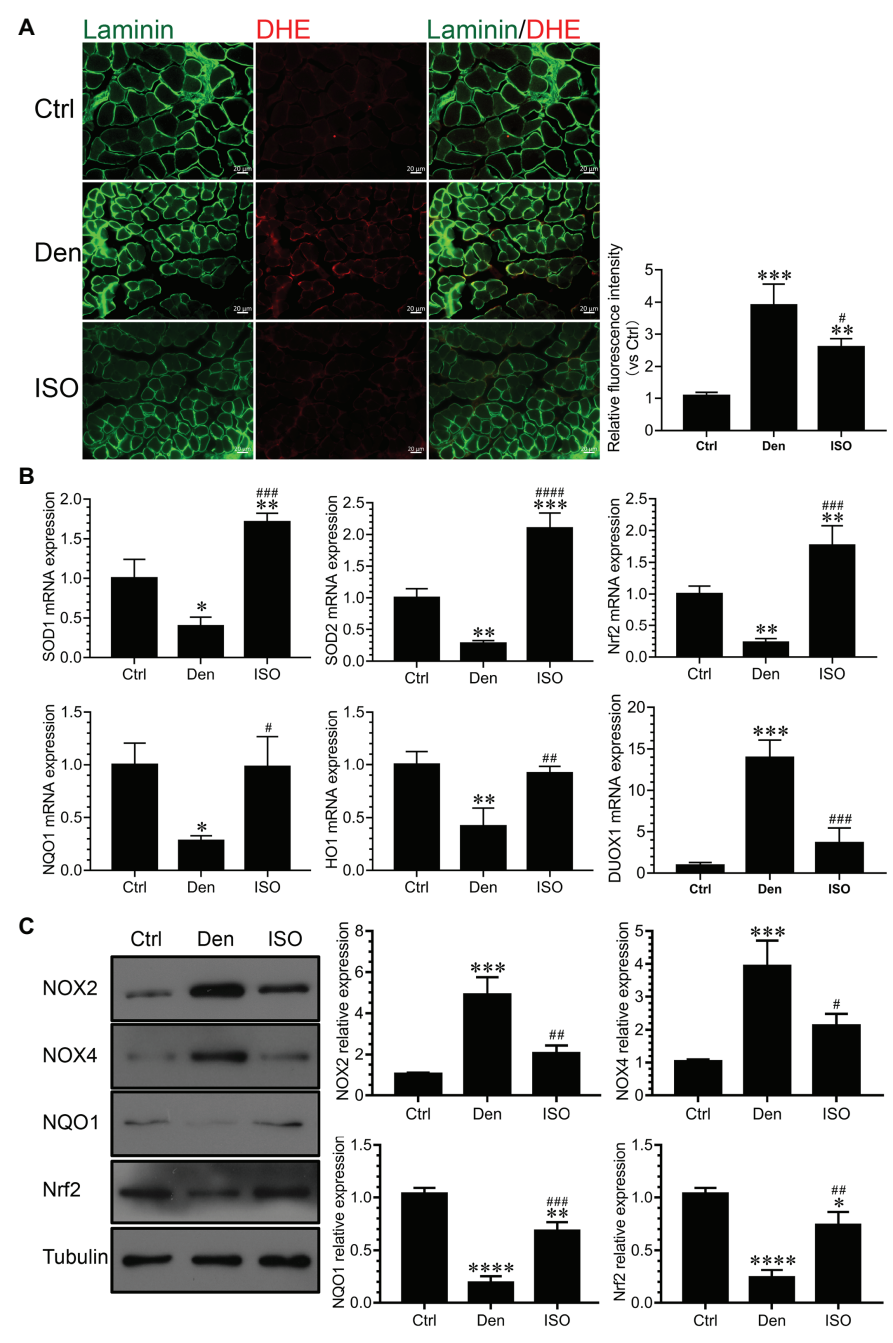
Isoquercitrin inhibits oxidative stress in denervated soleus muscles (*n* = 6). **(A)** Fluorescence diagram of reactive oxygen species (ROS) level and quantification of relative fluorescence intensity of each group in the dihydroethidium (DHE) probe detection experiment. **(B)** Quantitative polymerase chain reaction detection of the relative expression of antioxidant-related genes and ROS production-related genes. **(C)** Western blot of ROS-related and antioxidant-related proteins and quantification of the relative expressions. ROS, reactive oxygen species; Ctrl, control group; Den, denervation group; ISO, denervated target muscle plus isoquercitrin (20 mg/kg/d) group. ^*^p < 0.05, ^**^p < 0.01, ^***^p < 0.001, and ^****^p < 0.0001 vs. Ctrl; ^#^*p* < 0.05, ^##^*p* < 0.01, ^###^*p* < 0.001, and ^####^*p* < 0.0001 vs. Den.

Studies have reported that high ROS levels can cause proteolysis, muscle cell apoptosis, and eventual skeletal muscle atrophy (Theilen et al., 2017; Ábrigo et al., 2018; Guigni et al., 2018). Our previous study has suggested that excessive ROS causes oxidative stress damage, subsequently inducing the production of numerous inflammatory factors that cause inflammation (Shen et al., 2019). In the present study, we explored whether isoquercitrin can suppress the inflammatory response by suppressing oxidative stress. We used DHE probe detection to demonstrate that the ROS level in denervated target muscles was significantly higher than that in the control group and that isoquercitrin intervention could significantly inhibit the increase of ROS level in denervated target muscles (Figures 6A,B). qPCR and western blot findings showed that the expression of antioxidant-related genes and proteins (SOD1, SOD2, NRF2, NQO1, and HO1) in denervated target muscles significantly decreased and that of genes related to ROS production (Nox2, Nox4, and DUOX1) in denervated target muscles significantly increased. Isoquercitrin treatment significantly reversed the expression of antioxidant-related genes and proteins (SOD1, SOD2, NRF2, NQO1, and HO1) and ROS production-related genes (Nox2, Nox4, and DUOX1) in denervated target muscles (Figure 6C). These results suggested that isoquercitrin relieves denervated muscle atrophy by inhibiting oxidative stress and reducing inflammation.

## Discussion

Denervated muscle atrophy is a highly prevalent disease in clinical settings. Although several studies have been performed on denervated muscle atrophy and substantial progress has been made, there is no sound method for its prevention and treatment. The molecular mechanism of denervated muscle atrophy remains unelucidated (Cao et al., 2018). Currently, drug therapy for muscular atrophy mainly focuses on inhibiting protein degradation and promoting protein synthesis (Dutt et al., 2015). However, during the muscle atrophic process, there are several pathways involved in proteolysis and protein synthesis, such as the ubiquitin-proteasome system, autophagy-lysosome system, cathepsin hydrolysis, and insulin-like growth factor 1/phosphatidylinositide 3-kinase/protein kinase B-mediated anabolic pathways (Ma et al., 2019; Han et al., 2020; Nguyen et al., 2020). It is impractical to sequentially intervene in these pathways. If we can identify the early upstream trigger factors that concurrently regulate these pathways, interventions in these pathways may achieve better results.

Our previous study has shown that denervated muscle atrophy can be divided into oxidative stress, inflammatory, atrophic, and atrophic fibrosis stages. Because oxidative stress and inflammation can be sequentially activated within 24 h following skeletal muscle denervation, oxidative stress and inflammatory signals may play an essential early triggering role in denervated skeletal muscle atrophy (Shen et al., 2019). Therefore, the present study explored whether isoquercitrin, which has antioxidative and anti-inflammatory activities, can alleviate denervated muscle atrophy. Our findings confirm the role of oxidative stress and inflammation in denervated muscle atrophy and provide a scientific basis for isoquercitrin as a protective drug for the prevention and treatment of muscle atrophy. Furthermore, our study revealed that isoquercitrin inhibits target muscle atrophy following denervation, which is mainly characterized as inhibiting the reduction of the wet weight ratio and muscle fiber cross-sectional area of the denervated target muscle. The ubiquitin-proteasome system plays an important role in various muscle atrophies, including those associated with denervation, disuse, tumor cachexia, diabetes, and various chronic inflammatory conditions (Gao et al., 2018; Lala-Tabbert et al., 2019; Han et al., 2020; Nguyen et al., 2020). MAFbx and MuRF1 are two muscle-specific E3 ubiquitin ligases that are increased in various muscle atrophies and are confirmed as suitable markers of muscle atrophy (Bodine and Baehr, 2014). In the present study, isoquercitrin inhibited the increase in the expression of MAFbx and MuRF1 in denervated target muscles and reduced the degradation of MyHC, thereby alleviating denervated muscle atrophy. These results suggest that isoquercitrin relieves denervated muscle atrophy by inhibiting the ubiquitin-proteasome proteolytic system. Bandyopadhaya et al. (2019) reported that ROS accumulation may trigger the activity of the ubiquitin ligases MuRF1 and MAFbx. The expression of MAFbx and MuRF1 was inhibited by increase in the activity of the antioxidant factor HO1 in sepsis-induced muscle wasting (Yu et al., 2018a). Inflammation can induce muscle atrophy by enhancing the expression of MAFbx and MuRF1 (Hahn et al., 2020; Kim et al., 2020). TNF-α stimulates the expression of MAFbx in the skeletal muscle *via* the p38 MAPK pathway and promotes muscle fiber proteolysis (Ma, 2010). We observed that isoquercitrin can upregulate the expression of antioxidant factors (SOD1, SOD2, NRF2, NQO1, and HO1), downregulate the expression of ROS production-related factors (Nox2, Nox4, and DUOX1), inhibit the production of IL-1β, IL-6, and TNF-α, and inactivate JAK/STAT3 signaling. Therefore, isoquercitrin may reduce the expression of MAFbx and MuRF1 by inhibiting oxidative stress and inflammatory responses and further reduce MyHC degradation, thereby alleviating denervated muscle atrophy.

Mitophagy refers to the process that selectively removes damaged or incomplete mitochondria *via* the mechanism of autophagy. In the human body, mitophagy maintains the integrity of mitochondrial function, thereby delaying aging and treating diseases (Wing et al., 2011). Gatica et al. (2018) showed that maintaining a balance in autophagy is particularly important in the body and that excessive autophagy can cause skeletal muscle atrophy. Another study indicated a significant increase in the expression of beclin 1 and LC3B in the skeletal muscle in spinal muscular atrophy (Sandri, 2010). Autophagy plays an important role in the process of spinal muscular atrophy and denervated muscle atrophy. Autophagy inhibition can effectively relieve muscle atrophy, and autophagy is a key target for the treatment of muscle atrophy (Piras et al., 2017; Cicardi et al., 2019; Wang et al., 2019; Cui et al., 2020). Our findings demonstrate that isoquercitrin can significantly inhibit mitophagy caused by denervation of the target muscle, accompanied by decreased expression of the autophagy-related proteins ATG7, BNIP3, and PINK1. Therefore, isoquercitrin treatment may prevent the occurrence of a process associated with autophagy inhibition as a part of its protective effect against denervated muscle atrophy. ROS may induce autophagy by activating the mucolipin-lysosome Ca^2+^-transcription factor EB pathway (Zhang et al., 2016). Smuder et al. (2018) found that diaphragmatic mitochondrial ROS production during mechanical ventilation is essential to promote the expression of autophagy-related genes (such as *LC3*, *Atg7*, *Atg12*, *Beclin1*, and *p62*) and to increase the activity of cathepsin L, i.e., oxidative stress stimulates autophagy enhancement. In patients with chronic inflammation, inflammatory factors (IL-6, TNF-α, and TGF-β) can cause exacerbated mitophagy and dysfunction by reducing PGC-1α and upregulating autophagy-related genes (LC3B, Beclin-1, p62, Atg5, and Bnip3; Carson et al., 2016; VanderVeen et al., 2017). Our findings indicate that isoquercitrin can upregulate the expression of antioxidant-related genes in the target muscle following denervation, downregulate the expression of ROS production-related genes, inhibit inflammatory factors, and block the JAK/STAT3 pathway. Overall, isoquercitrin may inhibit autophagy in the target muscle following denervation by suppressing ROS and inflammatory signals, thereby inhibiting the expression of autophagy-related genes, reducing autophagy, and eventually protecting denervated muscle against atrophy.

Skeletal muscle comprises various fibers with different metabolic characteristics. Each muscle fulfills a specific function and responds differently to external stimuli and disturbances due to different innervations and fiber types (Lang et al., 2018). The conversion between muscle fiber types is a dominant feature of muscle atrophy. The slow-to-fast fiber type conversion severely impacts the function of the target muscle following denervation. In the present study, we found that isoquercitrin can delay the conversion from slow-to-fast muscle fibers following denervation, accompanied by a decrease in the expression of TnI-FS and an increase in the expression of TnI-SS. PGC-1α is a key factor that regulates mitochondrial function, modulates muscle fiber type, and controls fast-to-slow muscle fiber type conversion (Lin et al., 2002). Our findings revealed that isoquercitrin significantly inhibits the decreased expression of PGC-1α in denervated target muscles. Therefore, isoquercitrin may reverse the slow-to-fast fiber type conversion in denervated target muscles by promoting the expression of PGC-1α, thereby protecting against denervated muscle atrophy. Existing studies have shown that ROS production suppresses silent information regulator 1 (SIRT1)/PGC-1α signaling and that a decrease in ROS production can increase the expression of SIRT1 and PGC-1α proteins in the skeletal muscle of aging rats (Yu et al., 2018b; Yang et al., 2019). Moreover, inflammatory factors (IL-6, TNF-α, and TGF-β) can reduce the expression of PGC-1α (Carson et al., 2016; VanderVeen et al., 2017). The results obtained in our study indicate that isoquercitrin can inhibit oxidative stress and inflammation. Therefore, isoquercitrin can reduce autophagy and may inhibit oxidative stress and inflammation signals to promote the expression of PGC-1α following skeletal muscle denervation, by reversing the slow-to-fast fiber type conversion following denervation, thereby protecting against denervated muscle atrophy.

It should be mentioned that there are some limitations. In this study, we only studied the protective effect of isoquercetin on denervated soleus (slow-twitch fibers) muscle atrophy. Future studies to evaluate the effect of isoquercitrin on other muscles [e.g., TA (fast-twitch fibers) muscle] against denervation-induced muscle atrophy in mice are warranted. Pharmacokinetic study of isoquercitrin in plasma after intraperitoneal administration is a matter of significance, as well as the impact of isoquercitrin on contralateral leg. The young mice used in the experiments grew considerably during the period 14 days post nerve injury, which might induce some added impact compared to adult mice. Another drawback of our study was that all the analyses were done after 2 weeks of treatment, which do not allow to fine tune the molecular events underlying the process.

**Figure 7:**
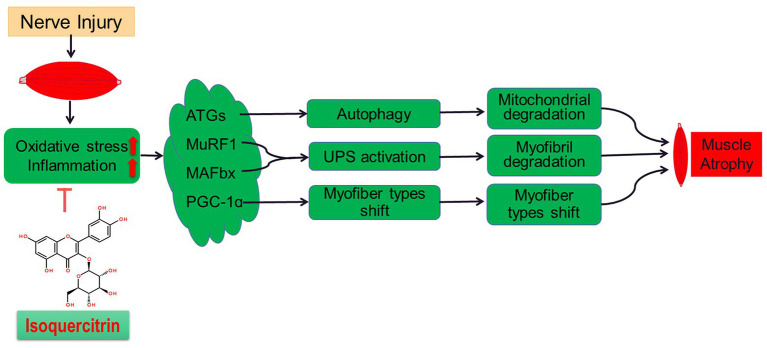
A schematic diagram illustrating the proposed mechanism by which peripheral nerve injury induces soleus muscle atrophy. Denervation-induced skeletal muscle atrophy is associated with oxidative stress and inflammation. The inhibition of oxidative stress and inflammation through Isoquercitrin alleviated denervation-induced skeletal muscle atrophy by reducing proteolysis, inhibiting mitophagy, and reversing the slow-to-fast fiber type conversion following denervation.

In summary, this study further confirms the role of oxidative stress and inflammatory response in denervated muscle atrophy. Isoquercitrin can alleviate denervated muscular atrophy by inhibiting oxidative stress and reducing inflammatory response to reduce autophagy, inhibit proteolysis *via* the ubiquitin-protease system, and suppress muscle fiber type conversion (Figure 7). Our findings enrich the knowledge regarding the molecular regulation mechanism of denervated muscular atrophy and provide a scientific basis for the use of isoquercitrin as a protective drug for the prevention and treatment of muscle atrophy.

## Data Availability Statement

The original contributions presented in the study are included in the article/Supplementary Material, further inquiries can be directed to the corresponding authors.

## Ethics Statement

The animal study was reviewed and approved by the animal care guidelines of Nantong University and ethically approved by Jiangsu Administration Committee of Experimental Animals.

## Author Contributions

HS and FD designed the study. YS, ZH, QZ, WM, XY, and JZ performed the experiments. YS, ZH, QZ, WM, XY, and JQ collected and assembled data. ZH and WM performed data analysis. FD provided scientific expertise. HS wrote the manuscript. All authors contributed to the article and approved the submitted version.

### Conflict of Interest

The authors declare that the research was conducted in the absence of any commercial or financial relationships that could be construed as a potential conflict of interest.

## Abbreviations

DHE: Dihydroethidium
ELISA: Enzyme-linked immunosorbent assay
ICR: Institute of Cancer Research
MAPK: Mitogen-activated protein kinase
MyHC: Myosin heavy chain
PBS: Phosphate-buffered saline
PCR: Polymerase chain reaction
PVDF: Polyvinylidene difluoride
ROS: Reactive oxygen species
TBST: Tris-buffered saline with Tween
TEM: Transmission electron microscopy

## References

[ref1] Ábrigo J. Campos F. Simon F. Riedel C. Cabrera D. Vilos C.. (2018). TGF-β requires the activation of canonical and non-canonical signalling pathways to induce skeletal muscle atrophy. Biol. Chem. 399, 253–264. 10.1515/hsz-2017-0217, PMID: 29140787

[ref2] Arouche-Delaperche L. Allenbach Y. Amelin D. Preusse C. Mouly V. Mauhin W.. (2017). Pathogenic role of anti-signal recognition protein and anti-3-hydroxy-3-methylglutaryl-CoA reductase antibodies in necrotizing myopathies: myofiber atrophy and impairment of muscle regeneration in necrotizing autoimmune myopathies. Ann. Neurol. 81, 538–548. 10.1002/ana.24902, PMID: 28224701

[ref3] Bandyopadhaya A. Tzika A. A. Rahme L. G. (2019). *Pseudomonas aeruginosa* quorum sensing molecule alters skeletal muscle protein homeostasis by perturbing the antioxidant defense system. mBio 10, e02211–e02219. 10.1128/mBio.02211-19, PMID: 31575771 PMC6775459

[ref4] Beehler B. C. Sleph P. G. Benmassaoud L. Grover G. J. (2006). Reduction of skeletal muscle atrophy by a proteasome inhibitor in a rat model of denervation. Exp. Biol. Med. 231, 335–341. 10.1177/153537020623100315, PMID: 16514182

[ref5] Bodine S. C. Baehr L. M. (2014). Skeletal muscle atrophy and the E3 ubiquitin ligases MuRF1 and MAFbx/atrogin-1. Am. J. Physiol. Endocrinol. Metab. 307, E469–E484. 10.1152/ajpendo.00204.2014, PMID: 25096180 PMC4166716

[ref6] Brzeszczyńska J. Meyer A. McGregor R. Schilb A. Degen S. Tadini V.. (2018). Alterations in the *in vitro* and *in vivo* regulation of muscle regeneration in healthy ageing and the influence of sarcopenia. J. Cachexia Sarcopenia Muscle 9, 93–105. 10.1002/jcsm.12252, PMID: 29214748 PMC5803613

[ref7] Cao R. Y. Li J. Dai Q. Li Q. Yang J. (2018). Muscle atrophy: present and future. Adv. Exp. Med. Biol. 1088, 605–624. 10.1007/978-981-13-1435-3_29, PMID: 30390273

[ref8] Carson J. A. Hardee J. P. VanderVeen B. N. (2016). The emerging role of skeletal muscle oxidative metabolism as a biological target and cellular regulator of cancer-induced muscle wasting. Semin. Cell Dev. Biol. 54, 53–67. 10.1016/j.semcdb.2015.11.005, PMID: 26593326 PMC4867246

[ref9] Castets P. Rion N. Théodore M. Falcetta D. Lin S. Reischl M.. (2019). mTORC1 and PKB/Akt control the muscle response to denervation by regulating autophagy and HDAC4. Nat. Commun. 10:3187. 10.1038/s41467-019-11227-4, PMID: 31320633 PMC6639401

[ref10] Ceco E. Weinberg S. E. Chandel N. S. Sznajder J. I. (2017). Metabolism and skeletal muscle homeostasis in lung disease. Am. J. Respir. Cell Mol. Biol. 57, 28–34. 10.1165/rcmb.2016-0355TR, PMID: 28085493 PMC5516279

[ref11] Cerquone Perpetuini A. Re Cecconi A. D. Chiappa M. Martinelli G. B. Fuoco C. Desiderio G.. (2018). Group I paks support muscle regeneration and counteract cancer-associated muscle atrophy. J. Cachexia Sarcopenia Muscle 9, 727–746. 10.1002/jcsm.12303, PMID: 29781585 PMC6104114

[ref12] Chiono V. Tonda-Turo C. (2015). Trends in the design of nerve guidance channels in peripheral nerve tissue engineering. Prog. Neurobiol. 131, 87–104. 10.1016/j.pneurobio.2015.06.001, PMID: 26093353

[ref13] Cicardi M. E. Cristofani R. Crippa V. Ferrari V. Tedesco B. Casarotto E.. (2019). Autophagic and proteasomal mediated removal of mutant androgen receptor in muscle models of spinal and bulbar muscular atrophy. Front. Endocrinol. 10:569. 10.3389/fendo.2019.00569, PMID: 31481932 PMC6710630

[ref14] Cui C. Han S. Tang S. He H. Shen X. Zhao J.. (2020). The autophagy regulatory molecule CSRP3 interacts with LC3 and protects against muscular dystrophy. Int. J. Mol. Sci. 21:749. 10.3390/ijms21030749, PMID: 31979369 PMC7037376

[ref15] Dai Y. Zhang H. Zhang J. Yan M. (2018). Isoquercetin attenuates oxidative stress and neuronal apoptosis after ischemia/reperfusion injury *via* Nrf2-mediated inhibition of the NOX4/ROS/NF-κB pathway. Chem. Biol. Interact. 284, 32–40. 10.1016/j.cbi.2018.02.017, PMID: 29454613

[ref16] Dumitru A. Radu B. M. Radu M. Cretoiu S. M. (2018). Muscle changes during atrophy. Adv. Exp. Med. Biol. 1088, 73–92. 10.1007/978-981-13-1435-3_4, PMID: 30390248

[ref17] Dutt V. Gupta S. Dabur R. Injeti E. Mittal A. (2015). Skeletal muscle atrophy: potential therapeutic agents and their mechanisms of action. Pharmacol. Res. 99, 86–100. 10.1016/j.phrs.2015.05.010, PMID: 26048279

[ref18] Ferreira D. M. S. Cheng A. J. Agudelo L. Z. Cervenka I. Chaillou T. Correia J. C.. (2019). LIM and cysteine-rich domains 1 (LMCD1) regulates skeletal muscle hypertrophy, calcium handling, and force. Skelet. Muscle 9:26. 10.1186/s13395-019-0214-1, PMID: 31666122 PMC6822430

[ref19] Gao Y. Arfat Y. Wang H. Goswami N. (2018). Muscle atrophy induced by mechanical unloading: mechanisms and potential countermeasures. Front. Physiol. 9:235. 10.3389/fphys.2018.00235, PMID: 29615929 PMC5869217

[ref20] Gatica D. Lahiri V. Klionsky D. J. (2018). Cargo recognition and degradation by selective autophagy. Nat. Cell Biol. 20, 233–242. 10.1038/s41556-018-0037-z, PMID: 29476151 PMC6028034

[ref21] Gueugneau M. d’Hose D. Barbé C. de Barsy M. Lause P. Maiter D.. (2018). Increased Serpina3n release into circulation during glucocorticoid-mediated muscle atrophy. J. Cachexia Sarcopenia Muscle 9, 929–946. 10.1002/jcsm.12315, PMID: 29989354 PMC6204594

[ref22] Guigni B. A. Callahan D. M. Tourville T. W. Miller M. S. Fiske B. Voigt T.. (2018). Skeletal muscle atrophy and dysfunction in breast cancer patients: role for chemotherapy-derived oxidant stress. Am. J. Physiol. Cell Physiol. 315, C744–C756. 10.1152/ajpcell.00002.2018, PMID: 30207784 PMC6293050

[ref23] Hahn A. Kny M. Pablo-Tortola C. Todiras M. Willenbrock M. Schmidt S.. (2020). Serum amyloid A1 mediates myotube atrophy *via* toll-like receptors. J. Cachexia Sarcopenia Muscle 11, 103–119. 10.1002/jcsm.12491, PMID: 31441598 PMC7015249

[ref24] Han Y. Lee H. Li H. Ryu J. H. (2020). Corylifol A from *Psoralea corylifolia L.* enhances myogenesis and alleviates muscle atrophy. Int. J. Mol. Sci. 21:1571. 10.3390/ijms21051571, PMID: 32106603 PMC7084366

[ref25] He Q. Qiu J. Dai M. Fang Q. Sun X. Gong Y.. (2016). MicroRNA-351 inhibits denervation-induced muscle atrophy by targeting TRAF6. Exp. Ther. Med. 12, 4029–4034. 10.3892/etm.2016.3856, PMID: 28101181 PMC5228305

[ref26] Higashino K. Matsuura T. Suganuma K. Yukata K. Nishisho T. Yasui N. (2013). Early changes in muscle atrophy and muscle fiber type conversion after spinal cord transection and peripheral nerve transection in rats. J. Neuroeng. Rehabil. 10:46. 10.1186/1743-0003-10-46, PMID: 23687941 PMC3668998

[ref27] Huang Z. Fang Q. Ma W. Zhang Q. Qiu J. Gu X.. (2019). Skeletal muscle atrophy was alleviated by salidroside through suppressing oxidative stress and inflammation during denervation. Front. Pharmacol. 10:997. 10.3389/fphar.2019.00997, PMID: 31616291 PMC6763704

[ref28] Hyatt H. Deminice R. Yoshihara T. Powers S. K. (2019). Mitochondrial dysfunction induces muscle atrophy during prolonged inactivity: a review of the causes and effects. Arch. Biochem. Biophys. 662, 49–60. 10.1016/j.abb.2018.11.005, PMID: 30452895 PMC6783132

[ref29] Janice Sánchez B. Tremblay A. K. Leduc-Gaudet J. P. Hall D. T. Kovacs E. Ma J. F.. (2019). Depletion of HuR in murine skeletal muscle enhances exercise endurance and prevents cancer-induced muscle atrophy. Nat. Commun. 10:4171. 10.1038/s41467-019-12186-6, PMID: 31519904 PMC6744452

[ref30] Jayachandran M. Wu Z. Ganesan K. Khalid S. Chung S. M. Xu B. (2019). Isoquercetin upregulates antioxidant genes, suppresses inflammatory cytokines and regulates AMPK pathway in streptozotocin-induced diabetic rats. Chem. Biol. Interact. 303, 62–69. 10.1016/j.cbi.2019.02.017, PMID: 30817903

[ref31] Kim J. Aydemir T. B. Jimenez-Rondan F. R. Ruggiero C. H. Kim M. H. Cousins R. J. (2020). Deletion of metal transporter Zip14 (Slc39a14) produces skeletal muscle wasting, endotoxemia, Mef2c activation and induction of miR-675 and Hspb7. Sci. Rep. 10:4050. 10.1038/s41598-020-61059-2, PMID: 32132660 PMC7055249

[ref32] Kim J. H. Lee S. Cho E. J. (2019). Acer okamotoanum and isoquercitrin improve cognitive function *via* attenuation of oxidative stress in high fat diet- and amyloid beta-induced mice. Food Funct. 10, 6803–6814. 10.1039/C9FO01694E, PMID: 31577306

[ref33] Lala-Tabbert N. Lejmi-Mrad R. Timusk K. Fukano M. Holbrook J. St-Jean M.. (2019). Targeted ablation of the cellular inhibitor of apoptosis 1 (cIAP1) attenuates denervation-induced skeletal muscle atrophy. Skelet. Muscle 9:13. 10.1186/s13395-019-0201-6, PMID: 31126323 PMC6533726

[ref34] Lang F. Khaghani S. Turk C. Wiederstein J. L. Holper S. Piller T.. (2018). Single muscle fiber proteomics reveals distinct protein changes in slow and fast fibers during muscle atrophy. J. Proteome Res. 17, 3333–3347. 10.1021/acs.jproteome.8b00093, PMID: 30142977

[ref35] Li J. Chan M. C. Yu Y. Bei Y. Chen P. Zhou Q.. (2017). miR-29b contributes to multiple types of muscle atrophy. Nat. Commun. 8:15201. 10.1038/ncomms15201, PMID: 28541289 PMC5458521

[ref36] Li J. Wang X. Wang Y. Lu C. Zheng D. Zhang J. (2019). Isoquercitrin, a flavonoid glucoside, exerts a positive effect on osteogenesis *in vitro* and *in vivo*. Chem. Biol. Interact. 297, 85–94. 10.1016/j.cbi.2018.10.018, PMID: 30365939

[ref37] Lin J. Wu H. Tarr P. T. Zhang C. Y. Wu Z. Boss O.. (2002). Transcriptional co-activator PGC-1 alpha drives the formation of slow-twitch muscle fibres. Nature 418, 797–801. 10.1038/nature00904, PMID: 12181572

[ref38] Livak K. J. Schmittgen T. D. (2001). Analysis of relative gene expression data using real-time quantitative PCR and the 2(-Delta Delta C(T)) method. Methods 25, 402–408. 10.1006/meth.2001.1262, PMID: 11846609

[ref39] Ma Q. (2010). Transcriptional responses to oxidative stress: pathological and toxicological implications. Pharmacol. Ther. 125, 376–393. 10.1016/j.pharmthera.2009.11.004, PMID: 19945483

[ref40] Ma W. Zhang R. Huang Z. Zhang Q. Xie X. Yang X.. (2019). PQQ ameliorates skeletal muscle atrophy, mitophagy and fiber type transition induced by denervation *via* inhibition of the inflammatory signaling pathways. Ann. Transl. Med. 7:440. 10.21037/atm.2019.08.101, PMID: 31700876 PMC6803183

[ref41] Milan G. Romanello V. Pescatore F. Armani A. Paik J. H. Frasson L.. (2015). Regulation of autophagy and the ubiquitin-proteasome system by the FoxO transcriptional network during muscle atrophy. Nat. Commun. 6:6670. 10.1038/ncomms7670, PMID: 25858807 PMC4403316

[ref42] Muller F. L. Song W. Jang Y. C. Liu Y. Sabia M. Richardson A.. (2007). Denervation-induced skeletal muscle atrophy is associated with increased mitochondrial ROS production. Am. J. Physiol. Regul. Integr. Comp. Physiol. 293, R1159–R1168. 10.1152/ajpregu.00767.2006, PMID: 17584954

[ref43] Nguyen T. T. N. Choi H. Jun H. S. (2020). Preventive effects of dulaglutide on disuse muscle atrophy through inhibition of inflammation and apoptosis by induction of Hsp72 expression. Front. Pharmacol. 11:90. 10.3389/fphar.2020.00090, PMID: 32153405 PMC7046759

[ref44] Piras A. Schiaffino L. Boido M. Valsecchi V. Guglielmotto M. De Amicis E.. (2017). Inhibition of autophagy delays motoneuron degeneration and extends lifespan in a mouse model of spinal muscular atrophy. Cell Death Dis. 8:3223. 10.1038/s41419-017-0086-4, PMID: 29259166 PMC5870600

[ref45] Qiu J. Fang Q. Xu T. Wu C. Xu L. Wang L.. (2018). Mechanistic role of reactive oxygen species and therapeutic potential of antioxidants in denervation- or fasting-induced skeletal muscle atrophy. Front. Physiol. 9:215. 10.3389/fphys.2018.00215, PMID: 29593571 PMC5861206

[ref46] Qiu J. Zhu J. Zhang R. Liang W. Ma W. Zhang Q.. (2019). miR-125b-5p targeting TRAF6 relieves skeletal muscle atrophy induced by fasting or denervation. Ann. Transl. Med. 7:456. 10.21037/atm.2019.08.39, PMID: 31700892 PMC6803201

[ref47] Quattrocelli M. Barefield D. Y. Warner J. L. Vo A. H. Hadhazy M. Earley J. U.. (2017). Intermittent glucocorticoid steroid dosing enhances muscle repair without eliciting muscle atrophy. J. Clin. Invest. 127, 2418–2432. 10.1172/JCI91445, PMID: 28481224 PMC5451235

[ref48] Reza M. M. Subramaniyam N. Sim C. M. Ge X. Sathiakumar D. McFarlane C.. (2017). Irisin is a pro-myogenic factor that induces skeletal muscle hypertrophy and rescues denervation-induced atrophy. Nat. Commun. 8:1104. 10.1038/s41467-017-01131-0, PMID: 29062100 PMC5653663

[ref49] Sakellariou G. K. McDonagh B. (2018). Redox homeostasis in age-related muscle atrophy. Adv. Exp. Med. Biol. 1088, 281–306. 10.1007/978-981-13-1435-3_13, PMID: 30390257

[ref50] Salucci S. Falcieri E. (2020). Polyphenols and their potential role in preventing skeletal muscle atrophy. Nutr. Res. 74, 10–22. 10.1016/j.nutres.2019.11.004, PMID: 31895993

[ref51] Sandri M. (2010). Autophagy in health and disease. 3. Involvement of autophagy in muscle atrophy. Am. J. Physiol. Cell Physiol. 298, C1291–C1297. 10.1152/ajpcell.00531.2009, PMID: 20089936

[ref52] Shen Y. Zhang R. Xu L. Wan Q. Zhu J. Gu J.. (2019). Microarray analysis of gene expression provides new insights into denervation-induced skeletal muscle atrophy. Front. Physiol. 10:1298. 10.3389/fphys.2019.01298, PMID: 31681010 PMC6798177

[ref53] Smuder A. J. Sollanek K. J. Nelson W. B. Min K. Talbert E. E. Kavazis A. N.. (2018). Crosstalk between autophagy and oxidative stress regulates proteolysis in the diaphragm during mechanical ventilation. Free Radic. Biol. Med. 115, 179–190. 10.1016/j.freeradbiomed.2017.11.025, PMID: 29197632 PMC5767544

[ref54] Sun H. Gong Y. Qiu J. Chen Y. Ding F. Zhao Q. (2014a). TRAF6 inhibition rescues dexamethasone-induced muscle atrophy. Int. J. Mol. Sci. 15, 11126–11141. 10.3390/ijms150611126, PMID: 24955790 PMC4100203

[ref55] Sun H. Li M. Gong L. Liu M. Ding F. Gu X. (2012). iTRAQ-coupled 2D LC-MS/MS analysis on differentially expressed proteins in denervated tibialis anterior muscle of *Rattus norvegicus*. Mol. Cell. Biochem. 364, 193–207. 10.1007/s11010-011-1218-2, PMID: 22227918

[ref56] Sun H. Liu J. Ding F. Wang X. Liu M. Gu X. (2006). Investigation of differentially expressed proteins in rat gastrocnemius muscle during denervation-reinnervation. J. Muscle Res. Cell Motil. 27, 241–250. 10.1007/s10974-006-9067-4, PMID: 16752196

[ref57] Sun H. Qiu J. Chen Y. Yu M. Ding F. Gu X. (2014b). Proteomic and bioinformatic analysis of differentially expressed proteins in denervated skeletal muscle. Int. J. Mol. Med. 33, 1586–1596. 10.3892/ijmm.2014.1737, PMID: 24715111

[ref58] Sun H. Zhu T. Ding F. Hu N. Gu X. (2009). Proteomic studies of rat tibialis anterior muscle during postnatal growth and development. Mol. Cell. Biochem. 332, 161–171. 10.1007/s11010-009-0186-2, PMID: 19554422

[ref59] Tajsharghi H. Oldfors A. (2013). Myosinopathies: pathology and mechanisms. Acta Neuropathol. 125, 3–18. 10.1007/s00401-012-1024-2, PMID: 22918376 PMC3535372

[ref60] Theilen N. T. Kunkel G. H. Tyagi S. C. (2017). The role of exercise and TFAM in preventing skeletal muscle atrophy. J. Cell. Physiol. 232, 2348–2358. 10.1002/jcp.25737, PMID: 27966783 PMC5444986

[ref61] Tos P. Ronchi G. Geuna S. Battiston B. (2013). Future perspectives in nerve repair and regeneration. Int. Rev. Neurobiol. 109, 165–192. 10.1016/b978-0-12-420045-6.00008-0, PMID: 24093612

[ref62] Tuffaha S. H. Singh P. Budihardjo J. D. Means K. R. Higgins J. P. Shores J. T.. (2016). Therapeutic augmentation of the growth hormone axis to improve outcomes following peripheral nerve injury. Expert Opin. Ther. Targets 20, 1259–1265. 10.1080/14728222.2016.1188079, PMID: 27192539

[ref63] VanderVeen B. N. Fix D. K. Carson J. A. (2017). Disrupted skeletal muscle mitochondrial dynamics, mitophagy, and biogenesis during cancer cachexia: a role for inflammation. Oxid. Med. Cell. Longev. 2017:3292087. 10.1155/2017/3292087, PMID: 28785374 PMC5530417

[ref64] Wang Y. Shao Y. Gao Y. Wan G. Wan D. Zhu H.. (2019). Catalpol prevents denervated muscular atrophy related to the inhibition of autophagy and reduces BAX/BCL2 ratio *via* mTOR pathway. Drug Des. Devel. Ther. 13, 243–253. 10.2147/DDDT.S188968, PMID: 30643390 PMC6319426

[ref65] Winbanks C. E. Murphy K. T. Bernardo B. C. Qian H. Liu Y. Sepulveda P. V.. (2016). Smad7 gene delivery prevents muscle wasting associated with cancer cachexia in mice. Sci. Transl. Med. 8:348ra398. 10.1126/scitranslmed.aac4976, PMID: 27440729

[ref66] Wing S. S. Lecker S. H. Jagoe R. T. (2011). Proteolysis in illness-associated skeletal muscle atrophy: from pathways to networks. Crit. Rev. Clin. Lab. Sci. 48, 49–70. 10.3109/10408363.2011.586171, PMID: 21699435 PMC5734931

[ref67] Wu C. Tang L. Ni X. Xu T. Fang Q. Xu L.. (2019). Salidroside attenuates denervation-induced skeletal muscle atrophy through negative regulation of pro-inflammatory cytokine. Front. Physiol. 10:665. 10.3389/fphys.2019.00665, PMID: 31293430 PMC6604664

[ref68] Xie W. Wang M. Chen C. Zhang X. Melzig M. F. (2016). Hepatoprotective effect of isoquercitrin against acetaminophen-induced liver injury. Life Sci. 152, 180–189. 10.1016/j.lfs.2016.04.002, PMID: 27049115

[ref69] Yamakawa H. Kusumoto D. Hashimoto H. Yuasa S. (2020). Stem cell aging in skeletal muscle regeneration and disease. Int. J. Mol. Sci. 21:1830. 10.3390/ijms21051830, PMID: 32155842 PMC7084237

[ref70] Yang W. Zhang G. Jiang F. Zeng Y. Zou P. An H.. (2019). BPDE and B[a]P induce mitochondrial compromise by ROS-mediated suppression of the SIRT1/TERT/PGC-1α pathway in spermatogenic cells both *in vitro* and *in vivo*. Toxicol. Appl. Pharmacol. 376, 17–37. 10.1016/j.taap.2019.05.004, PMID: 31085209

[ref71] Yin J. Yang L. Xie Y. Liu Y. Li S. Yang W.. (2018). Dkk3 dependent transcriptional regulation controls age related skeletal muscle atrophy. Nat. Commun. 9:1752. 10.1038/s41467-018-04038-6, PMID: 29717119 PMC5931527

[ref72] Yu X. Han W. Wang C. Sui D. Bian J. Bo L.. (2018a). Upregulation of heme oxygenase-1 by hemin alleviates sepsis-induced muscle wasting in mice. Oxid. Med. Cell. Longev. 2018:8927104. 10.1155/2018/8927104, PMID: 30533176 PMC6250022

[ref73] YuY.ZhaoY.TengF.LiJ.GuanY.XuJ.. (2018b). Berberine improves cognitive deficiency and muscular dysfunction *via* activation of the AMPK/SIRT1/PGC-1a pathway in skeletal muscle from naturally aging rats. J. Nutr. Health Aging 22, 710–717. 10.1007/s12603-018-1015-7, PMID: 29806860

[ref74] Zhang X. Yu L. Xu H. (2016). Lysosome calcium in ROS regulation of autophagy. Autophagy 12, 1954–1955. 10.1080/15548627.2016.1212787, PMID: 27485905 PMC5079666

[ref75] Zhu X. Kny M. Schmidt F. Hahn A. Wollersheim T. Kleber C.. (2017). Secreted frizzled-related protein 2 and inflammation-induced skeletal muscle atrophy. Crit. Care Med. 45, e169–e183. 10.1097/CCM.0000000000002056, PMID: 27661566

