# Supplementary material for: Isoquercitrin Delays Denervated Soleus Muscle Atrophy by Inhibiting Oxidative Stress and Inflammation
Source: Front Physiol. 2020 Aug 12;11:988. doi: 10.3389/fphys.2020.00988 (PMC7435639; doi:10.3389/fphys.2020.00988)
Supplement: Supplementary file 1 [file Data_Sheet_1.PDF]

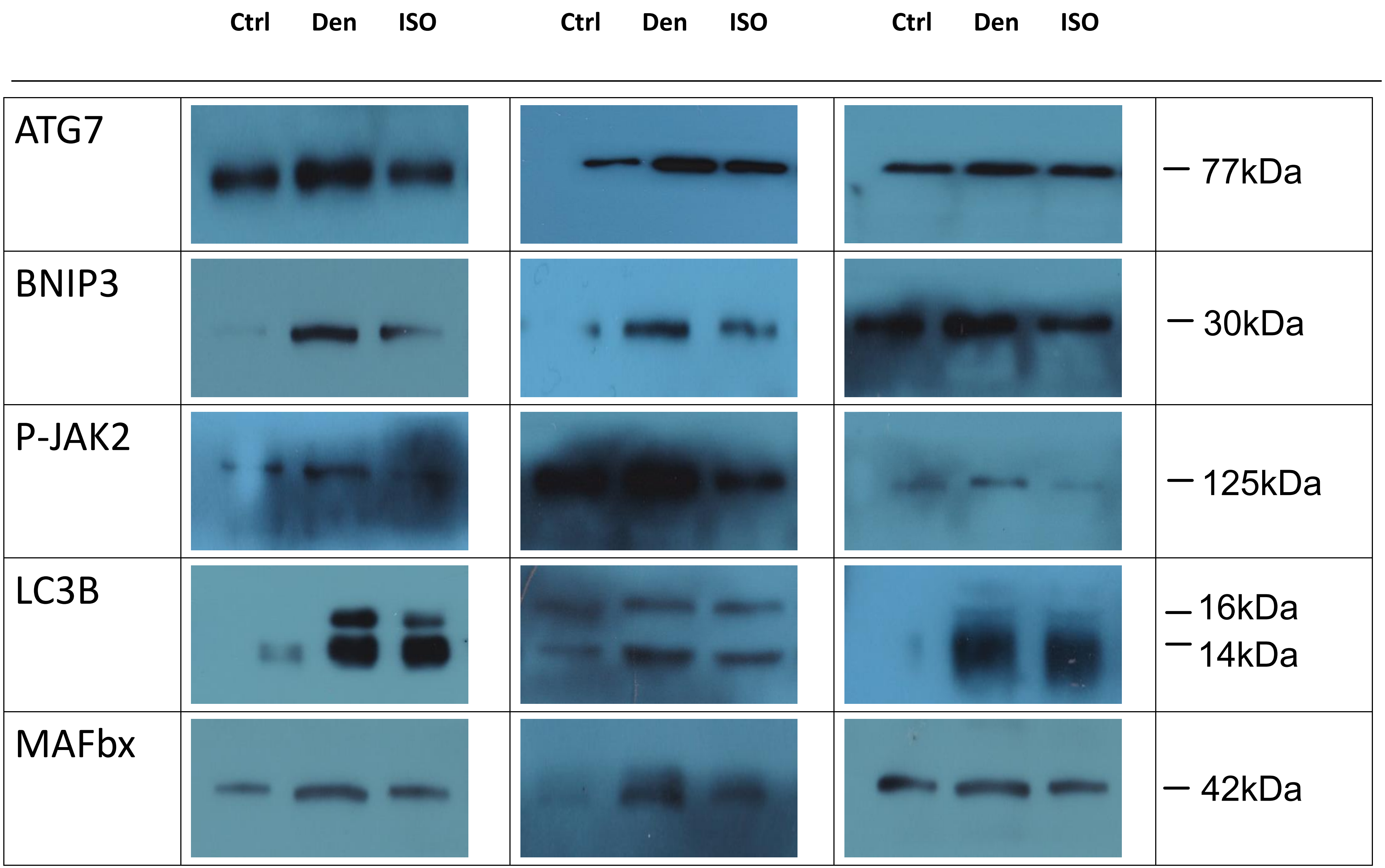
Ctrl
Den
ISO
Ctrl
Den
ISO
Ctrl
Den
ISO
ATG7
77kDa
BNIP3
30kDa
P-JAK2
125kDa
LC3B
16kDa
14kDa
MAFbx
42kDa

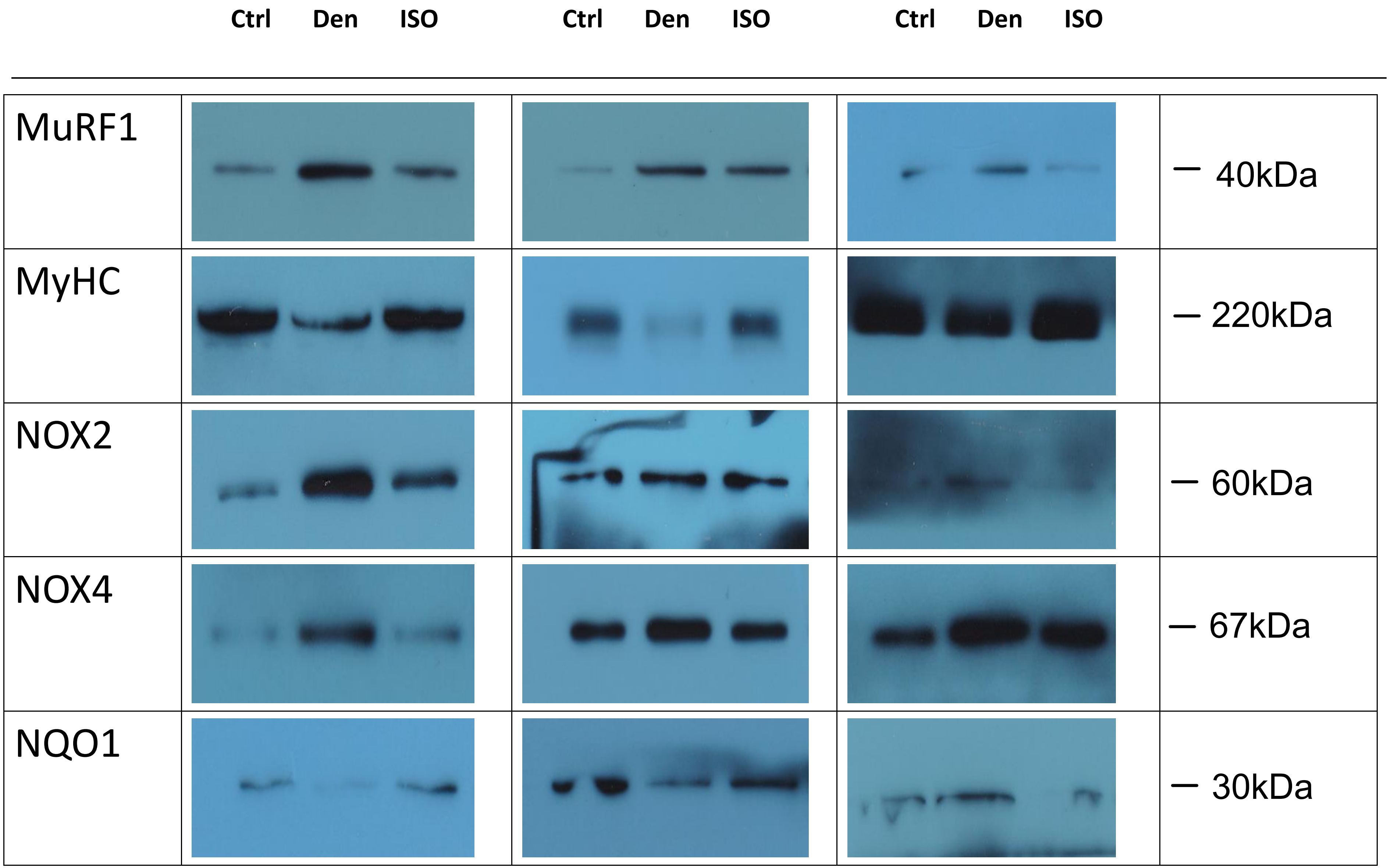
Ctrl Den ISO Ctrl Den ISO Ctrl Den ISO
MuRF1
MyHC
NOX2
NOX4
NQO1
40kDa
220kDa
60kDa
67kDa
30kDa

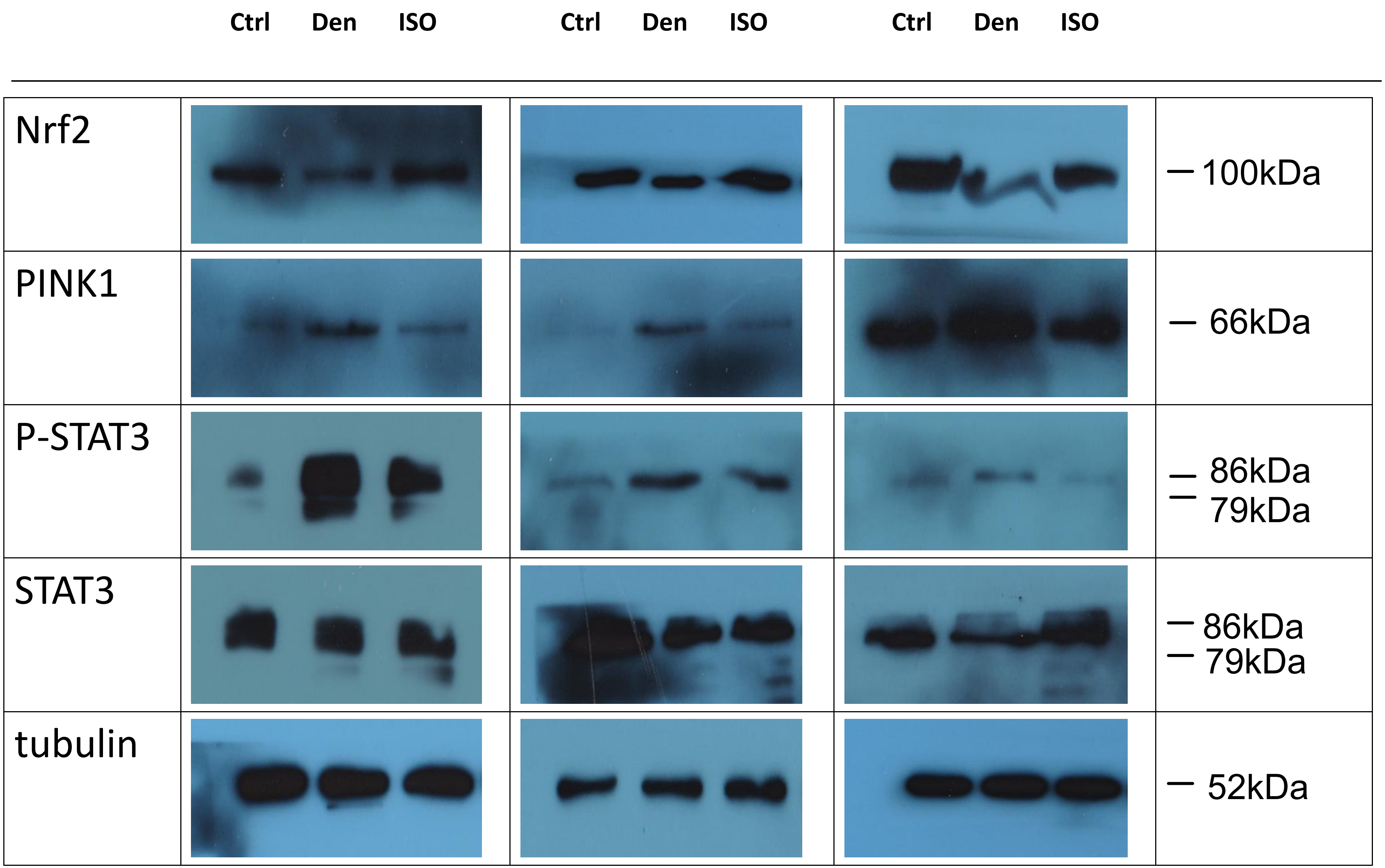
Ctrl
Den
ISO
Ctrl
Den
ISO
Ctrl
Den
ISO
Nrf2
PINK1
P-STAT3
STAT3
tubulin
100kDa
66kDa
86kDa
79kDa
86kDa
79kDa
52kDa

Ctrl **Den** **ISO** **Ctrl** **Den** **ISO** **Ctrl** **Den** **ISO**

Supplementary figure Western blot detection of related proteins. Ctrl: normal control group; Den: negative control (denervation) group; ISO: denervated target muscle plus isoquercitrin (20 mg/kg/d) group.
